# Targeting the inflammasome in Parkinson’s disease

**DOI:** 10.3389/fnagi.2022.957705

**Published:** 2022-10-12

**Authors:** Qi Su, Wei Lun Ng, Suh Yee Goh, Muhammad Yaaseen Gulam, Lin-Fa Wang, Eng-King Tan, Matae Ahn, Yin-Xia Chao

**Affiliations:** ^1^Programme in Emerging Infectious Diseases, Duke–NUS Medical School, Singapore, Singapore; ^2^Department of Neurology, Singapore General Hospital, Singapore, Singapore; ^3^Department of Research, National Neuroscience Institute, Singapore, Singapore; ^4^Neuroscience and Behavioural Disorders Program, Duke–NUS Medical School, Singapore, Singapore

**Keywords:** Parkinson’s disease, neuroinflammation, inflammasome, NLRP3, inhibitor

## Abstract

Parkinson’s disease (PD) is one of the most common neurodegenerative diseases in which neuroinflammation plays pivotal roles. An important mechanism of neuroinflammation is the NLRP3 inflammasome activation that has been implicated in PD pathogenesis. In this perspective, we will discuss the relationship of some key PD-associated proteins including α-synuclein and Parkin and their contribution to inflammasome activation. We will also review promising inhibitors of NLRP3 inflammasome pathway that have potential as novel PD therapeutics. Finally, we will provide a summary of current and potential *in vitro* and *in vivo* models that are available for therapeutic discovery and development.

## Introduction

Parkinson’s disease (PD) is the second-most common neurodegenerative disease, predominantly affecting the elderly. Its prevalence has increased more than 20% over the past 30 years (Ou et al., 2021). PD was first described by James Parkinson in 1817 and the neuropathological hallmark of PD includes substantial dopaminergic neuronal loss in the substantia nigra, leading to reduced dopamine level in the striatum (Lotharius and Brundin, 2002). Another key feature in the substantia nigra of PD patients is the presence of Lewy bodies in the neurons (Lotharius and Brundin, 2002). Clinical diagnosis depends on both motor symptoms such as resting tremor, rigidity, bradykinesia/akinesia and postural instability, and non-motor symptoms including orthostatic hypotension, constipation, urinary dysfunction, mood and sleep disorders, and cognitive impairment (Schrag et al., 2015; Williams-Gray and Worth, 2016). The pathogenesis of PD contributed by both environmental and genetic factors is rather complex and not fully understood (Kouli et al., 2018).

The limited treatment options also add to its socioeconomic impact. The use of dopamine precursor levodopa can be traced back to 1960 and it is still serving as a gold standard for PD treatment today, often combined with a peripheral decarboxylase inhibitor such as carbidopa (Fahn et al., 2004). Other pharmacological agents include dopamine agonists such as ropinirole and pramipexole, and monoamine oxidase B inhibitors such as selegiline, which are more commonly used for patients with milder symptoms (Armstrong and Okun, 2020). By modulating dopamine levels or its receptor activity, these agents improve motor symptoms for the early-stage PD patients. However, they are associated with significant side effects and do not help with alleviating non-motor symptoms. For instance, levodopa treatment’s side effects include orthostatic hypotension, gastrointestinal symptoms and hallucination as well as motor fluctuation and dyskinesia (Armstrong and Okun, 2020). Other approaches, including deep brain stimulation, can help medication-resistant symptoms to some extent, but also come with many side effects (Groiss et al., 2009; Kalia et al., 2013). Importantly, to date, no therapies are able to prevent or delay the disease progression. Thus, there is an enormous need to have a deeper understanding of PD pathogenesis and develop novel therapeutic approaches to improve the lives of PD patients.

Among the various novel approaches to manage PD, immunomodulation has gained much popularity recently. This approach is conceived based on the heavy involvement of the immune system in the pathogenesis and progression of PD. Neuroinflammation is one of the immune processes of paramount importance in PD (Wang et al., 2015). Reactive microglia increased significantly in the substantia nigra region of PD patients upon *post-mortem* examinations (McGeer et al., 1988; Roca et al., 2011). Moreover, enhanced microglial activation was also observed in various PD animal models, including α-synuclein overexpression models, as well as 1-methyl-4-phenyl-1,2,3,6-tetrahydropyridine (MPTP), 6-hydroxydopamine (6-OHDA) and rotenone neurotoxin-induced mice, rats and monkeys (Roca et al., 2011). The activated microglia led to elevated pro-inflammatory cytokines production in the midbrain region, including interleukin-1β (IL-1β), IL-6, IL-12, tumor necrosis factor-α (TNF-α), and other stress-inducing molecules such as reactive oxygen species (ROS) and nitric oxide (Sawada et al., 2006). Though microglial activation does have essential housekeeping roles such as removing neuronal cell debris, the chronic overactivation of microglia can cause overproduction of the pro-inflammatory cytokines and dopaminergic neuron degeneration (Ferrari et al., 2006; McCoy et al., 2006). This process is also self-amplifying as the ATP, α-synuclein, and metalloproteinase-3 (MMP-3) released from degenerated neurons further activate microglia, amplify neuroinflammation, and accelerate the neurodegeneration process (Hammond et al., 2019; Kelly et al., 2020). Besides central nervous system (CNS) inflammation, peripheral inflammation is also believed to play a pivotal role in PD. Peripheral pro-inflammatory stimuli can be transported to the brain, activate the primed microglia, prompt neuroinflammation, and exacerbate disease progression (Ferrari and Tarelli, 2011; Tan E.-K. et al., 2020; Tan J. S. Y. et al., 2020). CD4^+^ T lymphocytes and inflammatory cytokines including IL-1β, IL-6, IFN- γ, and TNF- α were found to be upregulated in the serum of PD patients as well (Brodacki et al., 2008; Harms et al., 2013).

One key component of neuroinflammation is inflammasome activation. The inflammasome pathways consist of sensor proteins, such as NOD-like receptors NLRP1, NLRP3, NLRC4, and AIM2-like receptors AIM2, IFI16, that recognize exogenous microbe-derived stimuli or endogenous molecular stress stimuli (Zheng et al., 2020). Once activated, these sensors will induce the activation of the intracellular adapter apoptosis-associated speck-like protein containing a CARD (ASC) which also contains a PRYIN domain (PYD). The ASC monomers then aggregate and form speck-like polymers that activate caspase-1. Activated caspase-1 induces pyroptotic cell death and promotes the release of inflammatory cytokine IL-1β that is capable of further amplifying inflammation (Dick et al., 2016; Zheng et al., 2020). Out of all the discovered inflammasome pathways, NLRP3 is the most well-studied. NLRP3 contains a PRYIN domain (PYD), a nucleotide binding and oligomerization domain (NACHT), an N-terminal caspase activation and recruitment domain (CARD), and a C-terminal leucine-rich repeats (LRRs) (Proell et al., 2008; Howe et al., 2013). The NLRP3 inflammasome pathway is heavily involved in many acute infections and chronic inflammatory diseases including PD. Due to the importance of NLRP3 in the PD pathogenesis and it being a fairly recent discovery, this work aims to explore the involvement of NLRP3 in PD and summarize some of the therapeutic agents targeting NLRP3. We will also evaluate the current and potential *in vitro* and *in vivo* PD models for studying pathogenesis and developing new therapeutics.

## Inflammasome activation in Parkinson’s disease pathogenesis

Inflammation activation in PD has been reported in PD patients as well as multiple *in vitro* and *in vivo* PD models. Clinical evidence obtained, *via* post-mortem examinations of PD patients’ brains, also showed increased NLRP3 inflammasome expression and activation at the substantia nigra where dopaminergic neuronal loss has occurred (Gordon et al., 2018; von Herrmann et al., 2018; Anderson et al., 2021). Interestingly, multiple NLRP3 inflammasome-related proteins, including NLRP3, ASC, caspase-1, and IL-1β, as well as α-synuclein proteins were detected in PD patient’s serum or plasma (Lin et al., 2017; Fan et al., 2020; Anderson et al., 2021). These circulating inflammasome-related proteins might be just a reflection of intracranial neuroinflammation or potentially a sign of a more complex cross-talk between peripheral and central inflammation. A link between NLRP3 inflammasome activation in microglia and the progression of both dopaminergic neurodegeneration and α-synuclein accumulation has been established in different PD mouse models. In 6-OHDA-induced PD mouse models, NLRP3 inflammasome was involved in dopaminergic neuronal loss and motor symptoms (Gordon et al., 2018). In MPTP mouse model, NLRP3 inflammasome activation in microglia was also shown to play a key role in pathogenesis of PD (Lee et al., 2019).

The key known molecular mechanism of NLRP3 activation in PD involves α-synuclein. α-synuclein aggregates or fibrils trigger a delayed but robust activation of NLRP3 inflammasome in mouse primary microglia, resulting in IL-1β secretion and ASC release without proptosis (Gordon et al., 2018). Similarly, α-synuclein fibrils can activate NLRP3 in human primary microglia (Pike et al., 2021). In human induced pluripotent stem cell (hiPSC)- derived microglia (hiMG), dual stimulation of Toll-like receptor 2 (TLR2) engagement and mitochondrial damage is implicated in the NLRP3 activation (Trudler et al., 2021). In addition, priming *via* TLR2 and activation *via* phagocytosis followed by ROS production and cytosolic cathepsin B release are demonstrated in human peripheral monocytes (Codolo et al., 2013). Although microglial endocytosis of and subsequent lysosomal cathepsin B release of α-synuclein is shown in BV2 cells, a murine microglial cell line, the role of TLRs, ROS and lysosome destabilization in NLRP3 activation in primary microglia is yet to be validated (Zhou et al., 2016).

Several links between several PD-associated genes and inflammasomes have also been reported. Although most PD cases are idiopathic, about 5–10% of them are hereditary and are attributed to autosomal recessive (such as *parkin/PARK2* and *PINK1/PARK6*) or autosomal dominant (such as *LRRK2*, *SNCA*, *VPS35*) mutations (Deng et al., 2018). Both Parkin and PINK1 seem to be negative regulators of NLRP3 inflammasome (Mouton-Liger et al., 2018). Loss of function mutation of Parkin leads to exacerbation of NLRP3 activation in blood-derived macrophages *via* induction of A20 (Mouton-Liger et al., 2018). More recently, this E3 ubiquitin ligase Parkin is shown to negatively regulate NLRP3 in dopamine neurons *via* ubiquitinating NLRP3 for proteasomal degradation and reducing mitochondrial-derived ROS production (Panicker et al., 2022).

Importantly, loss of Parkin activity observed in the context of both hereditary and sporadic PD models leads to neuronal NLRP3 assembly and cell death, while inhibition of NLRP3 inflammasome in neurons alleviates dopamine neuron degeneration. This suggests neuronal NLRP3 activation, independent of microglia, contributes to pathogenesis of PD. On the other hand, the kinase activity of LRRK2 is required for activation of NLRC4 inflammasome instead of NLRP3, through directly interacting with NLRC4 (Denes et al., 2015). Although NLRC4 has not shown to be involved in PD pathogenesis, it has been implicated in neuroinflammation in the context of acute brain injury (Denes et al., 2015). It is yet to find out how LRRK2 mutations affect NLRC4 activation and if modulation of NLRC4 contribute to the LRRK2-driven PD.

As new evidence evolves to understand the pathogenesis of PD, the gut-brain axis, a bidirectional network of signaling pathways which consists of multiple connections has gained increased interest. Enteric bacteria have been reported to modulate inflammatory pathways *via* NLRP3 signaling and ultimately influence brain homeostasis (Rutsch et al., 2020). A significant change in the composition of gut microbiota can in turn mitigate an inflammatory cascade *via* the gut-brain axis. This include alterations in compositions in several bacterial families such as Prevotellaceae, Verrucomicrobiaceae, Bradyrhizobiaceae, and Lactobacillaceae (Scheperjans et al., 2015). PD specific intestinal flora was observed to be starved off butyrate producing bacteria such as Lachnospiraceae that has anti-inflammatory properties (Keshavarzian et al., 2015). During dysbiosis, gut microbiota metabolites and products, such as tryptophan (Rothhammer et al., 2018). SCFAs, vitamins or neurotransmitters, may translocate into the bloodstream and subsequently to the brain with altered permeability of the blood-brain barrier (BBB) and activate CNS inflammatory cells including microglia (Luczynski et al., 2016; Pellegrini et al., 2020). These products can also activate peripheral immune cells which release cytokines or migrate to the CNS and affect brain physiology (Shi et al., 2017; Pellegrini et al., 2020). The NLRP3 inflammasome plays a key role in this gut-brain communication as it is a sentinel sensor of the enteric bacteria. Recent study showed that NLRP3 gene deficiency in mice altered the gut microbiota composition and affected both mood-related behaviors and locomotor activities, whether this is also true in Parkinson’s disease has yet to be studied (Zhang Y. et al., 2019). Exposure to environmental toxins such as herbicides/pesticides, MPTP, and heavy metals like manganese was also associated with Parkinsonism (Ballard et al., 1985; Gorell et al., 1997, 1998). A few of these agents, including rotenone and MPTP, are now commonly used to induce PD in established animal models.

**FIGURE 1:**
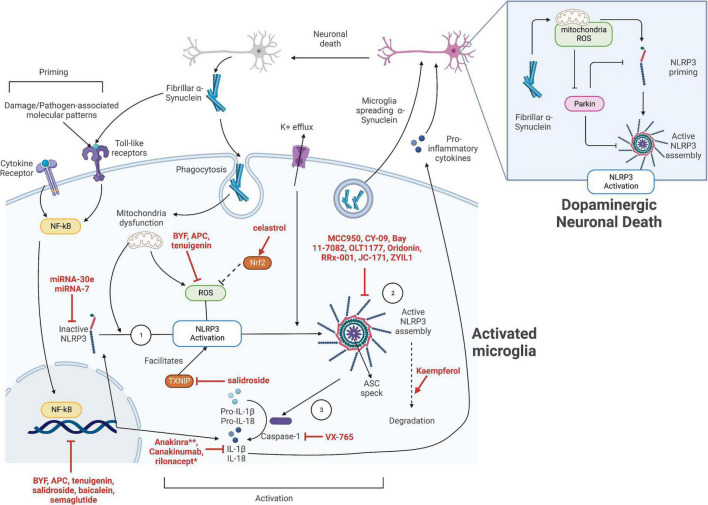
The strategies for inhibition of NLRP3 inflammasome activation in Parkinson’s disease (PD). The activation of NLRP3 inflammasome in microglia can be divided into the priming stage and activation stage. Various inflammatory cytokines and damage-associated or pathogen-associated molecular patterns, including fibrillar α-synuclein, can activate the NF-κB pathway to upregulate the expression of NLRP3 sensor protein, pro-IL-1β, and pro-IL-18. The inactive NLRP3 proteins then oligomerize upon activation by the various stimuli including potassium ion efflux, mitochondrial dysfunction, and related reactive oxygen species (ROS) release. These signals might be generated by, but not limited to, the phagocytosis of fibrillar α-synuclein. The activated NLRP3 proteins then trigger an assembly of apoptosis-associated speck-like protein containing a CARD (ASC), which subsequently activate pro-caspase 1. Activated caspase 1 facilitates the maturation of pro-cytokines, leading to pro-inflammatory cytokine release. The released cytokines as well as fibrillar α-synuclein directly exocytosed by the microglia promotes neuronal death, leading to the release of more fibrillar α-synuclein, thus amplifying microglial activation and neuroinflammation. Potential target sites of NLRP3 activation in microglia include: ➀ targeting the NF-κB priming pathway to prevent the upregulation of NLRP3 and cytokines, however, this might be highly non-specific; ➁ directly targeting the inflammasome components and preventing its activation; ➂ targeting the downstream inflammasome effectors. The specific NLRP3 inhibitors mentioned in this work are indicated in red. **Anakinra and rilonacept indirectly inhibit IL-1β by binding to IL-1 receptors and serving as a decoy receptor for IL-1β, respectively.

These evidences suggest that the NLRP3 inflammasome-driven neuroinflammation plays a critical role in the pathogenesis of neuroinflammation in PD. Therefore, microglial and neuronal NLRP3 inflammasome is a promising target for PD drug development with great potential. Figure 1 summarizes the molecular mechanisms of α-synuclein-mediated NLRP3 inflammasome activation in PD as well as the potential drug targets.

## Anti-inflammasome as a novel therapeutic approach for Parkinson’s disease

Since the NLRP3 inflammasome pathway plays a pivotal role in PD pathogenesis, targeting NLRP3 can be a viable approach to develop novel therapeutics to slow down the disease progression. Table 1 summarizes the inhibitors targeting NLRP3 inflammasome that have been investigated in pre-clinical or clinical studies, especially in the context of PD and other CNS diseases. Figure 1 indicates the mechanisms of action for the NLRP3 inhibitors discussed in this section. These NLRP3 inhibitors with an effect in CNS diseases indicate their ability to cross blood-brain barrier (BBB) and hence are promising drug candidates for PD.

**TABLE 1:** Direct NLRP3 inhibitors as potential Parkinson’s disease (PD) therapeutics at pre-clinical stages.

Target category	Inflammasome component	Direct NLRP3 inhibitors	Mechanism of inhibition	Current CNS models
Sensor	NLRP3	MCC950	Blocks ATPase activity Locks NLRP3 in inactive state (Coll et al., 2015, 2019)	MPTP-induced mice (Huang et al., 2021) α-synuclein PFF–injected mice (Gordon et al., 2018) MitoPark mice (Gordon et al., 2018) 6-OHDA-lesioned mice (Gordon et al., 2018)
		CY-09	Blocks ATPase activity Binds NACHT region of NLRP3 (Jiang W. et al., 2017)	Stroke mice (Sun et al., 2020)
		Bay 11-7082	Blocks ATPase activity (Juliana et al., 2010) Unknown binding site	Spinal cord injury mice (Jiang W. et al., 2017)
		OLT1177	Blocks ATPase activity (Marchetti et al., 2018) Unknown binding site	Alzheimer’s disease mice (Lonnemann et al., 2020) Experimental autoimmune encephalomyelitis (Sanchez-Fernandez et al., 2019)
		Oridonin	Attenuates NLRP3-NEK7 interaction Binds Cys279 (NACHT domain) (He et al., 2018)	Traumatic brain injury mice (Zhao et al., 2022)
		RRx-001	Attenuates NLRP3-NEK7 interaction Binds Cys409 (NACHT domain) (Chen Y. et al., 2021)	Experimental autoimmune encephalomyelitis mice (Chen Y. et al., 2021)
		JC-171	Attenuates NLRP3-ASC (Guo et al., 2017)	Experimental autoimmune encephalomyelitis (Guo et al., 2017)
Effectors	Caspase-1	VX-765	Reduce caspase-1-induced α-synuclein aggregation (Wang et al., 2016)	Multiple system atrophy (MSA) mice (Bassil et al., 2016) Alzheimer mice (Flores et al., 2020)
	Gasdermin D	Dimethyl fumarate	Succinates C191 Blocks GSDMD oligomerization (Yadav et al., 2021)	Experimental autoimmune encephalomyelitis mice (Yadav et al., 2021)

### Direct NLRP3 inhibitors

MCC950 is a NLRP3-specific small molecular inhibitor that directly interacts the ATP-hydrolysis motif within NACHT domain, blocks its ATPase activity and locks NLRP3 in an inactive conformation (Coll et al., 2015, 2019). It is the most widely used tool for NLRP3 inhibition in research and has been tested in a wide range of NLRP3-driven disease models including *in vitro* and *in vivo* PD models. The *in vitro* IC_50_ of MCC950 is 7.5 nM in mice bone marrow derived macrophages and 8.1 nM in human monocyte derived macrophages (Coll et al., 2015). The small molecule is able to penetrate the blood-brain barrier and achieve a concentration higher than the IC50 in the CNS (Gordon et al., 2018). Upon administration, MCC950 inhibited fibrillar α-synuclein-induced NLRP3 activation and reduced dopaminergic neuron degeneration, aggregation of α-synuclein, and the motor symptoms experienced by the PD mice (Gordon et al., 2018). In addition, MCC950 derivative, ZYIL1 is currently under phase I safety trial and also under clinical investigation in Cryopyrin Associated Periodic Syndrome patients (clinical trial identifiers: NCT04972188 and NCT05186051).

Though MCC950 seems to be a promising candidate for further development, its phase II clinical trial for rheumatoid arthritis was discontinued due to its hepatotoxicity (Mangan et al., 2018). Besides MCC950, several NLRP3 specific inhibitors have been investigated in CNS disease model through interfering NLRP3 domain and binding sites. This suggests that NLRP3 inhibitors are capable of crossing blood-brain barrier and exerts its inflammasome inhibition activity in neurodegeneration context. For example, CY-09 appears to inhibit ATPase activity and binds to Walker A site in NACHT domain of NLRP3 and is shown to reduce neuroinflammation in cerebral ischemic stroke model (Jiang H. et al., 2017; Sun et al., 2020). Additionally, several compounds that show ATPase blocking activity with unidentified binding sites was investigated and proven to be effective in CNS diseases. Some of the inhibitors include Bay-11-7082 (spinal cord injury) (Juliana et al., 2010; Jiang H. et al., 2017) and OLT1177 (AD, EAE) (Marchetti et al., 2018; Sanchez-Fernandez et al., 2019; Lonnemann et al., 2020). Besides direct NLRP3 inhibition, compounds that modifies key domain and affects protein-protein interaction of NLRP3 were also identified in neuronal diseases. This is demonstrated through Oridonin (TBI) (He et al., 2018; Zhao et al., 2022) and RRx-001 (EAE) (Chen Y. et al., 2021), in which both disrupts NLRP3-NEK7 interaction while JC-171 (EAE) attenuates NLRP3-ASC interaction (Guo et al., 2017). The disruption of JC-171 on NLRP3-ASC interaction was shown in reduced ASC level pulldown by NLRP3 with co-immunoprecipitation assay (Guo et al., 2017). Though Oridonin and RRx-001 share similar mechanism in blocking NLRP3-NEK7, they covalently modify C279 and C409 in the NACHT domain in NLRP3, respectively (Chen Y. et al., 2021).

ASC is an adaptor that acts downstream of NLRP3 and activate Caspase-1. However, little is known about the regulation of ASC oligomerization and currently no inhibitor is discovered.

### Inhibitors targeting downstream effectors and cytokines

After NLRP3 oligomers are formed upon external stimulation, it allows recruitment of adaptor protein ASC which subsequently promote downstream Caspase-1 activation and cleavage of Gasdermin D effectors, both of which are critical steps in proinflammatory components release from the cells. Thus, targeting downstream effectors of the NLRP3 inflammasome is an important strategy in controlling inflammation in PD.

#### Caspase-1

Besides facilitate IL-1β maturation and Gasdermin D cleavage, Caspase-1 also directly truncates α-synuclein molecules to a pro-aggregation state (Wang et al., 2016). Subsequent aggregation of α-synuclein lead to neuronal death and neuroinflammation. The use of a promising Caspase-1 inhibitor VX-765 was shown to reduce α-synuclein aggregation in transgenic mice multiple system atrophy (MSA) model and reduce neuroinflammation in Alzheimer mice model (Bassil et al., 2016; Flores et al., 2020). Despite the lack of *in vivo* study using VX-765 on PD animal models, *in vitro* experiments in PD neuronal cell lines reported satisfactory neuroprotection (Wang et al., 2016). Intraperitoneal injection of VX-765 also reduced neuroinflammation in mice model of spinal cord injury, proving its ability to cross the BBB (Chen J. et al., 2021). VX-765 is currently undergoing clinical investigation in epilepsy (clinical trial identifier: NCT01048255) and psoriasis (clinical trial identifier: NCT00205465) without any reports of toxicity yet. However, targeting caspase-1 is even less specific than targeting ASC as it is the universal effector of multiple inflammasomes.

#### Gasdermin D

Gasdermin D (GSDMD) is a key effector in NLRP3 inflammasome pathway which control pyroptosis and proinflammatory components release from the cells including IL-1β and IL18. Given that GSDMD modulates the release of IL-1β downstream of inflammasome activation, it is served as a pharmacological target to block cell lysis and proinflammatory contents and implicated in CNS disease. For instance, Dimethyl fumarate (DMF) succinates C191, block GSDMD oligomerization in autoimmune encephalitis (Yadav et al., 2021). However, GSDMD inhibitors targeting PD might be limited since DMF is suggested to affect infiltration of macrophages in the CNS rather than neurons (Yadav et al., 2021).

#### Interleukin-1β

Interleukin-1β is the pro-inflammatory cytokines released upon inflammasome activation and thus it can be a primary target in NLRP3 related pathology. There are three proteins approved for use in targeting IL-1β and the IL-1 receptors: Anakinra, Canakinumab, and rilonacept. Anakinra competes in binding to the IL-1 receptor, Canakinumab is a neutralizing antibody to IL-1β and rilonacept is a soluble decoy receptor that could bind both IL-1a and IL-1β, sequestering circulating IL-1 (Murray et al., 2015; Mangan et al., 2018). Nevertheless, the IL-1β inhibitors remains a limited options for PD treatment due to the poor penetrating ability at the blood-brain barrier (Murray et al., 2015; Mangan et al., 2018).

### Indirect inhibitors of NLRP3

Apart from direct NLRP3 inhibition, several other compounds are shown to regulate NLRP3 indirectly *via* modulating its transcription, inflammasome priming, protein expression or post-translational modifications. Therefore, these compounds suppress NLRP3 activity and IL-1β release independently of their direct effect on any components in the NLRP3 inflammasome.

For example, a natural flavanol, Kaempferol degrades NLRP3 through autophagy and ubiquitin related proteolysis which protects dopaminergic neuron loss in PD mice model (Han X. et al., 2019). Besides, various indirect NLRP3 inhibitors isolated from traditional Chinese medicine regulates NLRP3 *via* NF-kb pathway: Bushen-Yizhi Formula (BYF), Antrodia camphorate polysaccharide (APC), tenuigenin, salidroside, and baicalein (Fan et al., 2017; Mo et al., 2018; Han C. et al., 2019; Rui et al., 2020; Zhang et al., 2020). Next, a recombinant peptide semaglutide, an analogs of GLP-1, was shown to have neuroprotective effects in MPTP induced mice model *via* inhibiting NLRP3 through NF-kb signaling (Zhang L. et al., 2019; Chen X. et al., 2021). Another novel approach in inhibiting NLRP3 is microRNA which affects protein expression *via* post-transcriptional regulation. MicroRNA-30e and microRNA-7 mimics are two of the leading candidates demonstrated to effectively and specifically target the expression of NLRP3 (Zhou et al., 2016; Li et al., 2018). The levels of inflammatory cytokines, including IL-1β, IL-18, and TNF-α, were also lowered due to the miRNA-30e agomir-mediated decrease in NLRP3 expression (Li et al., 2018). In a similar manner, miRNA-7 was found to inhibit NLRP3 expression in both MPTP-induced PD mice and α-synuclein-overexpression transgenic mice (Zhou et al., 2016). The resulting dampening in caspase 1 activation and IL-1β production lead to a neuroprotective effect on the dopaminergic neurons in both models (Zhou et al., 2016).

NLRP3 driven neuroinflammation has been found to be critical in neurodegenerative diseases. Though some of the NLRP3 inhibitors are entering early clinical stages, more investigations are required to robustly test the NLRP3 inhibitors efficacy and discover new inhibitors targeting inflammasome pathway. Given that the involvement of inflammasome NLRC4, AIM2, and NLRP1 in diseases is emerging, molecules that block multiple inflammasome is a promising therapeutic strategy, for instance inhibiting ASC formation. Hence, targeting multiple layers of inflammasome will be a promising next generation anti-inflammatory approach to effectively control excessive inflammation.

## *In vitro* models of Parkinson’s disease

The discovery and development of novel PD therapeutics strongly rely on the presence of robust *in vitro* models. Though many *in vitro* models have been developed over the past few decades, the study of NLRP3 in PD introduces an additional challenge on the existing models as the mechanism involves a complex interplay between the immune system and the neuronal system. Currently available *in vitro* PD models can be categorized into four groups: immortalized cell lines, primary cells, induced pluripotent stem cell (iPSC) lines, and organoids. Human embryonic kidney cells (HEK293) and human neuroglioma cells (H4) are commonly used for PD studies due to the ease of culture and manipulation (Tabrizi, 2000; McLean et al., 2001). However, they are non-neuronal cell types and do not intrinsically express the inflammasome components to effectively study the effect of NLRP3 manipulation in PD. Pheochromocytoma (P12) cells were derived from rat adrenal medulla with the expression of NLRP3 inflammasome pathway components (Malagelada and Greene, 2008; Gong et al., 2018). However, PC12’s non-human origin may lead to altered intracellular signaling. Co-cultures of microglia and/or astrocytes with neuronal cell lines, such as the human neuroblastoma SH-SY5Y cells and the Lund human mesencephalic (LUHMES) cells, have also been employed to closely mimic the physiological environment of neurons in the human brain, providing a more relevant system to study the effect of microglia NLRP3 activation (Sherer et al., 2002; Zhang et al., 2014). However, these cell lines are hard to manage and manipulate, leading to high variabilities. In addition, the neoplastic origin of SH-SY5Y does not represent the true characteristic and physiology of primary neurons. Besides immortalized cell lines, primary cells are also used in PD studies. Primary dopaminergic neurons and primary cortical neurons are commonly used due to their similar characteristics to human neurons in the physiological environment (Freundt et al., 2012; Gaven et al., 2014). However, primary neuronal cell culture’s usage is also limited by the great variabilities imposed by the differences in host species and the efficiencies in specific cell type isolation. The primary neuronal cells also do not accurately reflect the actual physiology *in vivo*.

Moving beyond traditional cell line models, iPSC and 3D organoids have gained much popularity. Both of these models can be patient-derived and thus could carry the specific gene mutations from the hosts to achieve more precise reconstruction of the disease genotype (Martínez-Morales and Liste, 2012; Chlebanowska et al., 2020). In particular, iPSCs have the potential to differentiate into any cell types including neurons, astrocytes and microglia, which can be extremely difficult to obtain directly from the patients (Badanjak et al., 2021). However, iPSC may not be the best model for age-related diseases since the cellular hallmarks of aging are reversed during reprogramming (Mertens et al., 2018; Strässler et al., 2018). In addition, the clonal heterogeneity of the iPSC derived from the same donor makes it difficult to define meaningful phenotypic differences (Devine et al., 2011). Thus, clonal selection or more specific gene editing techniques are required to generate better iPSC-based models. The 3D human organoids, especially the midbrain organoids, have unique advantages of being a 3D structure containing a mixture of cells including dopaminergic neurons, astrocytes, and microglia, closely mimicking the *in vivo* environment (Galet et al., 2020). However, the development of organoids can be highly time-consuming with a high degree of variability. Though organoid models attempt to recapitulate the cellular composition of *in vivo* brain, few organoid models actually include microglia, which is of paramount importance in the study of NLRP3 in PD (Ormel et al., 2018).

Despite the various limitations, the value of *in vitro* PD models cannot be overlooked. Established immortalized/primary cell models, iPSCs and organoids can be used for large scale screening and validation of potential drug candidates with higher efficiency and reproducibility (Skibinski and Finkbeiner, 2011; Jo et al., 2016). In addition, since peripheral inflammation plays an important role in the pathogenesis and progression of PD, it can be easily and robustly modeled by *in vitro* cells such as human peripheral blood mononuclear cells (PBMCs). Having effective *in vitro* models of peripheral inflammation/inflammasome activation may provide a new angle in the therapeutics and intervention discovery for PD.

## *In vivo* models of Parkinson’s disease

Though *in vitro* models provide significant convenience and streamlining for PD research, the research on PD pathogenesis and the screening for potential therapeutics cannot be substantiated and validated without robust PD animal models. The common PD animal models involve the use of neurotoxins such as MPTP (Dovero et al., 2016), 6-OHDA (Sauer and Oertel, 1994), and pesticides/herbicides including rotenone, paraquat, and maneb (Domico et al., 2006; Chia et al., 2020; Cristóvão et al., 2020; Innos and Hickey, 2021). Besides chemical inducers, transgenic animal models are also used to investigate the phenotypes of specific mutations in PD-related genes such as PTEN-induced putative kinase 1 (PINK1), DJ-1, and Parkin (Kitada et al., 1998; Bonifati et al., 2003; Valente Enza et al., 2004). Overexpression of wild-type or variants of α-synuclein is also a strategy commonly explored to model the important role of α-synuclein in PD pathogenesis (Koprich et al., 2017).

The choice of model organism is also critical in evaluating the success of PD investigation. A systematic review has revealed that rodents are the most commonly used animals for both neurotoxin and transgenic PD models due to their ease of handling and conducting genetic manipulation as well as closely resembling the human anatomy (Kin et al., 2019). Non-rodents, such as the zebrafish, drosophila and *C. elegans*, are more commonly used as transgenic models than neurotoxin models in PD research because of their well-annotated genome and relatively shorter lifespan (Kin et al., 2019). One limitation of these organisms is that they do not intrinsically express α-synuclein. Although primates and other mammals, such as cats, dogs, and minipigs, are physiologically closer to humans, they are not as commonly used in PD research due to higher cost and increased difficulty in genetic manipulation and handling/breeding (Kin et al., 2019).

## Conclusion

Since NLRP3 inflammasome pathway activation contributes significantly to PD neuroinflammation, targeting NLRP3 as a PD therapeutic strategy is a promising approach that has been gaining increasing attention over the recent years. We discussed the key role of the PD-associated genes including α-synuclein and Parkin in NLRP3 activation and PD pathogenesis. We also systematically catalog the NLRP3 inflammasome inhibitors that have been tested in PD and other CNS disease models. Lastly, we highlighted the recent advances and limitations in PD *in vitro* and *in vivo* models which aid in future study design. These research tools are essential for future screening, validation, and development of novel therapeutics.

## Data availability statement

The original contributions presented in this study are included in the article/supplementary material, further inquiries can be directed to the corresponding authors.

## Author contributions

Y-XC, MA, WLN, and QS conceived the work. QS, MA, and WLN wrote the manuscript. SYG, MYG, L-FW, E-KT, and Y-XC reviewed and edited the manuscript. All authors have read and agreed to the published version of the manuscript.
